# Evaluation of bleaching efficacy and cytotoxicity of an experimental hydrogel containing MnO-Doped Biosilicate^®^ activated by violet LED light

**DOI:** 10.1590/1678-7765-2025-0503

**Published:** 2026-03-02

**Authors:** Rafael Dascanio, Rafael Antonio de Oliveira Ribeiro, Marina Trevelin Souza, Matheus Kury, Edgar Dutra Zanotto, Carlos Alberto de Souza Costa, Vanessa Cavalli

**Affiliations:** 1 Universidade Estadual de Campinas Faculdade de Odontologia de Piracicaba Departamento de Dentística Piracicaba SP Brasil Universidade Estadual de Campinas (UNICAMP), Faculdade de Odontologia de Piracicaba, Departamento de Dentística, Piracicaba, SP, Brasil.; 2 Universidade Estadual Paulista Faculdade de Odontologia de Araraquara Departamento de Materiais Dentários e Prótese Araraquara SP Brasil Universidade Estadual Paulista (UNESP), Faculdade de Odontologia de Araraquara, Departamento de Materiais Dentários e Prótese, Araraquara, SP, Brasil.; 3 Universidade Federal de São Carlos Departamento de Engenharia de Materiais Laboratório de Materiais Vítreos São Carlos SP Brasil Universidade Federal de São Carlos (UFSCar), Departamento de Engenharia de Materiais, Laboratório de Materiais Vítreos (LaMaV), São Carlos, SP, Brasil.; 4 Universidade Paulista Departamento de Dentística São Paulo SP Brasil Universidade Paulista (UNIP), Departamento de Dentística, São Paulo, SP, Brasil.; 5 Universidade Estadual Paulista Faculdade de Odontologia de Araraquara Departamento de Fisiologia e Patologia Araraquara SP Brasil Universidade Estadual Paulista (UNESP), Faculdade de Odontologia de Araraquara, Departamento de Fisiologia e Patologia, Araraquara, SP, Brasil.

**Keywords:** Hydrogen peroxide, Biosilicate^®^, Cytotoxicity

## Abstract

Although hydrogen peroxide (HP) at 35% is effective in dental bleaching, the cytotoxicity associated with its use remains a significant concern. In this context, this study aimed to develop a bleaching hydrogel containing 6% HP, Biosilicate^®^ (BioS) doped with manganese oxide (MnO_BioS), and irradiated with violet LED light (LED). Enamel/dentin discs were submitted to the following bleaching treatments (n=08): 35%HP (positive control), 6%HP gel containing BioS or MnO_BioS (0 and 10 wt%), with or without LED irradiation. The discs were adapted to artificial pulp chambers (APCs), with the enamel exposed for bleaching and the dentin facing the culture medium (DMEM). Bleaching was performed in three 30-minute sessions with 7-day intervals. After bleaching, the extracts (DMEM + bleaching gel components diffused through the discs) were collected and applied to the odontoblast-like MDPC-23 cells. Color change (ΔE_00_) and changes in the whiteness index (ΔWI_D_) were determined before (T_0_) and after the last bleaching session (T_2_). Cell viability (MTT, %), HP diffusion (µg/mL), oxidative cell stress (OxS), and cell fluorescence (live/dead assay, by confocal microscopy) were assessed after the first session (T_1_). The data were analyzed using ANOVA and Tukey’s test (p=0.05). The addition of 6%HP_MnO_BioS_LED exceeded ΔWI_D_ compared to 35%HP, showing statistical differences from the other groups, while ΔE_00_ was statistically similar to 35%HP. 6%HP_MnO_BioS_LED demonstrated higher cell viability, lower HP diffusion, and reduced oxidative stress (OxS) compared to the other groups (p<0.05). 6%HP_MnO_BioS_LED increased bleaching potential and presented lower cytotoxicity compared to 35%HP.

## Graphical abstract

**Figure:**
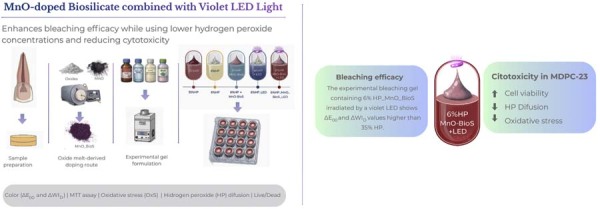

## Introduction

Despite the proven efficacy of in-office dental bleaching with high-concentration hydrogen peroxide (HP), the procedure can cause sensitivity in 50% to 93% of patients.^1^ This sensitivity is related to HP’s ability to diffuse through the enamel and dentin, triggering an inflammatory cascade that can damage the pulp tissue cells.^2^ This damage is associated with cellular oxidative stress, which intensifies as the diffusion of HP into the pulp environment increases, coming into contact with the cells.^1^ Furthermore, research conducted on healthy human teeth has shown that the use of high-concentration bleaching gels can cause areas of partial necrosis in the coronal pulp, along with inflammation in the surrounding tissues.^3^ This can lead to an increase in intrapulpal pressure and the stimulation of nerve fibers in the pulp, resulting in dental hypersensitivity. One strategy proposed to reduce these adverse effects has been the use of bleaching gels containing lower concentrations of HP.^4^ However, *in vitro* studies have shown that even at lower concentrations, HP bleaching still induces the release of reactive oxygen species (ROS), leading to oxidative stress in dental tissues, which may help explain the temporary sensitivity reported by patients in clinical settings.^5^ To enhance the bleaching efficacy of low-concentration HP gels while minimizing their cytotoxic effects, the incorporation of metal catalysts into the bleaching gel, such as manganese oxide (MnO), has shown to be promising.^6^ This element not only accelerates the decomposition of HP but also limits peroxide diffusion through the enamel–dentin complex, thereby minimizing oxidative stress and cellular cytotoxicity.^6,7^ The catalytic action of MnO₂ decreases the activation energy required for HP decomposition, leading to a faster and more controlled release of reactive oxygen species and water.^8^

Moreover, bioactive materials can play a synergistic role in minimizing the adverse effects of HP on both enamel/dentin structure and pulp, reducing demineralization and oxidative stress.^9^ One example is Biosilicate^®^ (BioS), which was initially introduced for the treatment of dentin hypersensitivity and has demonstrated the ability to release ions when in contact with bodily fluids.^10-12^ When incorporated into bleaching gels, BioS has shown potential to preserve the mineral content of enamel and reduce the trans-enamel-dentin diffusion of HP, thus minimizing oxidative stress and cellular cytotoxicity in enamel with initial erosion lesions.^9^

Still seeking to enhance the bleaching efficacy of low-concentration hydrogen peroxide (HP) gels, the use of violet-spectrum light sources (405–410 nm) stimulates HP decomposition and may alter the optical behavior of dental tissues.^13,14^ Moreover, when transition metal-based catalysts or metal-oxide photocatalysts are present, light activation can further accelerate radical generation and peroxide decomposition, enhancing bleaching outcomes.^15,16^ The light energy emitted can increase HP breakdown, generating a greater number of free radicals and other reactive species, thereby augmenting HP’s stain-removal capacity. The literature indicates that violet light can intensify hydroxyl radical (•OH) release either via photolysis — the light-induced dissociation of chemical compounds — or via thermocatalysis, where absorbed light is converted into heat, thus lowering activation energy and accelerating radical production.^13^

In a previous *in vitro* study, the incorporation of manganese oxide (MnO) into Biosilicate® (BioS) and its application in a bleaching hydrogel containing a reduced hydrogen peroxide (HP) concentration of 6%, activated by violet LED light, has shown promising results.^17^ This formulation led to a significant increase in the gel’s pH and produced a bleaching efficacy comparable to that of conventional 35% HP gels. Furthermore, the MnO-doped BioS maintained its bioactive potential, evidenced by an enhanced calcium-to-phosphate ratio and increased enamel surface microhardness after treatment.^17^ These findings suggested that the MnO_BioS system could combine esthetic performance with structural preservation of the dental substrate. However, the cytotoxic potential of this novel bleaching hydrogel on odontoblastic-like cells has not yet been elucidated. Therefore, assessing the biological safety of this innovative formulation is essential to confirm its translational potential. It is crucial to determine whether the MnO_BioS-modified bleaching gel can maintain its whitening efficacy while reducing cytotoxicity and oxidative stress, contributing to safer and more effective in-office bleaching protocols. This study evaluated the bleaching efficacy and cytotoxicity of a hydrogel containing MnO_BioS and 6% HP, irradiated with violet LED light. The null hypotheses were that the presence of MnO_BioS would not influence (1) the bleaching efficacy, (2) cell viability or oxidative stress in MDPC-23 odontoblastic cells, (3) trans-enamel–dentin HP diffusion, or (4) the cellular morphology after exposure to the hydrogel.

## Methodology

### Experimental design

Forty bovine enamel/dentin discs were prepared and selected based on their initial average color values (L* parameter). The discs were fixed in artificial pulp chambers (APCs) and randomly subjected to the treatments (n=8/group) as follows:

- 35%HP: 35% hydrogen peroxide (HP) gel (Whiteness HP, FGM, Indústria e Comércio de Produtos Odontológicos, Joinville, SC, Brazil).
- 6%HP: experimental 6% HP gel.
- 6%HP_MnO_BioS: experimental 6%HP gel with MnO-doped Biosilicate^®^ (BioS).
- 6%HP_LED: experimental 6%HP gel irradiated with violet LED light.
- 6%HP_MnO_BioS_LED: experimental 6%HP gel with MnO-doped BioS irradiated with violet LED light.

The bleaching treatment was performed in three 30-minute sessions, with and without violet light irradiation (20 cycles of 1 minute, 30-second intervals between irradiation cycles; 405 ± 10 nm, 1.2 W/cm^2^, emission window area = 10.7 cm^2^, Bright Max Whitening, MMO, São Carlos, SP, Brazil), according to the experimental groups. Between bleaching protocols, the enamel blocks were kept in artificial saliva (pH 7.0) at 37°C. Color change (ΔE_00_) and the whitening Index (ΔWI_D_) were determined before (T_0_) and after the last bleaching session (T_2_). The viability of MDPC-23 cells (MTT, %), trans-enamel/dentin diffusion of HP (µg/mL), cellular oxidative stress (OxS), and live/dead cell confocal fluorescence microscopy assays were evaluated immediately after the first bleaching session (T_1_).

### Sample preparation

Bovine incisors with intact enamel, free from fractures and fissures, were selected. The teeth were cleaned and disinfected in a 0.4% thymol solution (Labsynth, Diadema, SP, Brazil). Enamel/dentin discs (5 mm in diameter and 3 mm in thickness) were obtained from the central region of the bovine incisors using a benchtop drill (FSB 16, Pratika, Shulz, SP, Brazil). The dentin surface of each disc was polished with #600 grit sandpaper to ensure parallelism and achieve a standardized total thickness of 2.3 mm. The enamel/dentin specimens were stained with neutralized black tea (pH 7.0, adjusted with NaOH) for 48 hours and subsequently stored in artificial saliva for 7 days.^17^ The sample size (n=8 per group) was determined *a priori* using G*Power, considering color change (ΔE_00_) as the primary outcome variable. Data from a pilot study conducted by our group were used for this calculation, in which the control group (35% HP) exhibited a mean ΔE_00_ of 5.5 and the experimental group (6% HP + MnO_BioS + violet LED) a mean ΔE_00_ of 7.2, with a standard deviation of 2.5. Assuming a significance level of 5% (α=0.05) and a statistical power of 80% (β=0.20), the calculated effect size (0.35) indicated that eight specimens per group would be sufficient to detect significant differences between treatments.

### Synthesis of manganese oxide-doped Biosilicate® (MnO_BioS)

**Figure 1:**
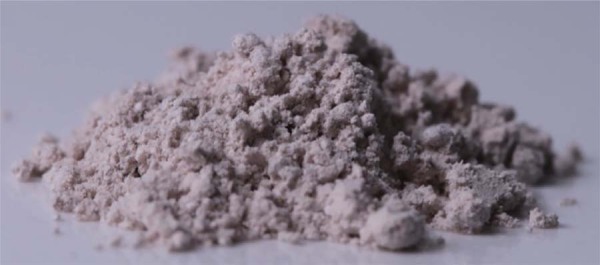
MnO-doped Biosilicate® (MnO_BioS) powder after agate milling in a planetary mill.

The Biosilicate^®^ (MnO_BioS) was synthesized using the oxide fusion route,^17^ incorporating 2.5 mg/mL of Manganese Oxide (MnO) adjusted according to the calcium proportion.^17^ Its composition was adjusted to 24.3Na₂O–26.9(xCaO-(1−x)MnO)–46.3SiO₂–2.5P₂O₅ (x=1 and 0.9, in weight %). First, the raw materials—CaCO₃, Na₂CO₃, SiO₂, NaH₂PO₄, and MnO—were mixed and homogenized in a jar mill for 24 hours. The mixture was then fused in a platinum crucible using a Bottom Load furnace (Nabertherm, DE) at 1450°C until a complete and homogeneous fusion was achieved. After fusion, the material was rapidly cooled to form a vitreous phase. MnO_BioS cylinders were obtained with a diameter of 12 mm and height of 50 mm. These cylinders were then ground again in a jar with agate milling media in a planetary mill (Fritsch, DE) until a mean particle size of 5 μm (d50) was achieved (Figure 1).

### Gel preparation

**Figure 2:**
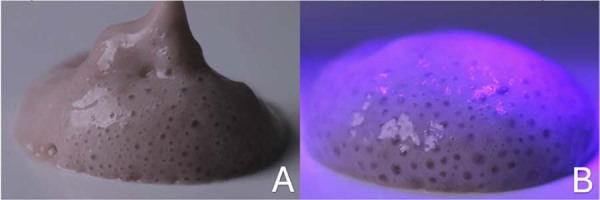
(2A) Hydrogel containing 6%HP and MnO_BioS after mixing in Speed Mixer. (2B) Hydrogel containing 6%HP and MnO_BioS irradiated with violet LED light.

The positive control bleaching gel consisted of 35%HP (Whiteness HP, FGM, Indústria e Comércio de Produtos Odontológicos, Joinville, SC, Brazil), prepared according to the manufacturer’s instructions. For the formulation of 6%HP, a dilution of the 35%HP was made, and then added to a thickening gel with carboxymethylcellulose (CMC) in the ratio of 0.4 g of the thickener to 0.54 mL of the 6%HP solution. The mixture was homogenized in a Speed Mixer (DAC Iso 1. FVZ, FlackTek, Inc., Hann, Germany) at 2,000 rpm for 2 minutes. In the experimental gels containing MnO_BioS (Figure 2A), CMC was used as a thickener combined with distilled water (H₂Od). The MnO_BioS particles, synthesized by the conventional fusion method, were incorporated into the CMC gel and homogenized under the same equipment and speed conditions. The gel-to-thickener ratio was kept identical to the 6%HP gel but with the presence of the particles.^17^ In Figure 2B, the 6%HP_MnO_BioS gel can be observed after irradiation with violet LED light. To ensure consistency with the previously cited *in vitro* study, the pH of all bleaching gels was measured prior to use under the same preparation conditions. The pH values obtained were 6.7 for 35%HP, 6.81 for 6%HP, and 9.32 for 6%HP_MnO_BioS, confirming the alkalinizing effect of MnO-doped Biosilicate^®^.

### Color analysis

A digital spectrophotometer (EasyShade, Vita Zahnfabrik, Bad Säckingen, Germany) was used to determine the color parameters L* (black-white axis), a* (red-green axis), and b* (yellow-blue axis). The color variation was evaluated using the CIEDE2000 formula (ΔE_00_)^18^:


$$\Delta E_{00}=\sqrt{{\left(\frac{\Delta L^{\prime }}{K_{L}SL}\right)}^{2}+{\left(\frac{\Delta C^{\prime }}{K_{C}SC}\right)}^{2}+{\left(\frac{\Delta H^{\prime }}{K_{H}SH}\right)}^{2}+RT\left(\frac{\Delta C^{\prime }}{K_{C}SC}\right)\left(\frac{\Delta H^{\prime }}{K_{H}SH}\right)}$$

The variation in the whiteness index was calculated using the formula:


$$WI_{D}=[(0.511\times L)-(2.324\times a*)-(1.100\times b*)]$$

ΔE_00_ and ΔWI_D_ were calculated after staining with black tea (T_0_) and 24 hours after the last bleaching session (T_2_). The ΔE_00_ values were evaluated based on the perception threshold (PT) and acceptance threshold (AT), set at 0.81 and 1.8 units, respectively. Similarly, the difference in the Whiteness Index (ΔWI_D_) were compared to the PT and AT thresholds of 0.7 and 2.6, respectively. These thresholds (50:50% PT and AT) are based on perception and acceptance criteria established by both professional and non-professional volunteers in prospective multicenter studies.^18^

### Specimen preparation in artificial pulp chambers (APCs)

The dental discs were adapted into artificial pulp chambers using two silicone rings, sealed with wax to prevent any leakage of the bleaching gel into the pulp space, according to the previously described protocol. The assembly, consisting of the chambers and discs, was sterilized using ethylene oxide. The disc-APC assemblies were placed into 24-well plates. Only the dentin surface was in contact with the culture medium, while the enamel surface was exposed to the bleaching protocol. Immediately after the bleaching procedures, the culture medium in contact with the dentin was collected, homogenized, and aliquoted into 24-well plates, where pulp cells had been previously cultured. The extracts were incubated for 1 hour in contact with the cells and immediately analyzed according to the methodology described.^5,9^

### MDPC-23 cell culture

The MDPC-23 odontoblastic cell line, immortalized and stored in liquid nitrogen, was thawed and cultured in 75 cm^2^ flasks. The pulp cells were maintained in culture medium using Dulbecco’s Modified Eagle Medium (DMEM), supplemented with 10% fetal bovine serum, 100 IU/mL penicillin, 100 µg/mL streptomycin, and 2 mmol/L glutamine. The plates containing the cells were maintained in a humidified atmosphere at 37°C with 5% CO₂ and 95% air. The cells were seeded and counted using an inverted microscope and subcultured as needed to achieve the required number of cells.^5,9^

### Cellular metabolism (MTT)

The APC was fixed in 24-well acrylic plates, ensuring direct contact with the dentin surface submerged in the culture medium. After the 30-minute bleaching protocol, the bleaching gels were completely removed. Then, cell viability was assessed based on the ability of active mitochondria to convert a tetrazolium compound into an insoluble product. Viable cells with succinate dehydrogenase activity produced blue formazan crystals. After bleaching, the MDPC-23 cells that adhered to the bottom of the plates were incubated in a solution composed of 90 μL of DMEM and 10 μL of MTT solution. The formazan crystals were dissolved, and absorbance was measured at 570 nm. The absorbance value of the groups without bleaching treatment was considered 100% cell viability, serving as a reference to calculate cell viability in the other experimental groups.^9^

### Oxidative stress (OxS) measurement

The cellular oxidative stress assay evaluated ROS production by the cells immediately after bleaching. For this, prior to exposure to the extracts, MDPC-23 cells were exposed for 30 minutes to 10 μg/mL of the carboxy-H2DCFDA dye. This dye has the ability to penetrate cells and, in the presence of ROS, is converted into a fluorescent compound, allowing quantification by flow cytometry. The cells were analyzed using a flow cytometer with a blue laser (488 nm). The data were analyzed with FlowJo software (Version 10.6.2) and expressed as a percentage of the untreated control group.^5,9^

### HP diffusion

The diffusion method was applied to determine the amount of HP that permeated the dentin, with dentin discs exposed to the culture medium (n=8/group). After 30 minutes of bleaching exposure, the samples were removed and immediately placed in 1 mL of H₂O₂, where they were incubated at 37°C. After 30 minutes, 100 μL of the solution was collected and subjected to a reaction with peroxidase (10 U/mL) in the presence of 3,3′,5,5′-tetramethylbenzidine (TMB, 1:10,000) for quantification, with absorbance measured at 650 nm.^5,9^

### Confocal microscopy with live/dead fluorescence

One hour after exposure to the extracts, MDPC-23 cells were subjected to the live/dead assay (live/dead cell viability/cytotoxicity kit, Invitrogen, CA, USA). The cells were incubated (n=8) for 30 minutes in contact with 100 µL of DMEM supplemented with Calcein AM and homodimeric ethidium marker at a 1:1000 concentration. The cells were then visualized under a fluorescence microscope (Leica DM 5500B, Nussloch GmbH, Germany) to observe the cellular patterns of each group.^9^

### Statistical analysis

Data were analyzed using SPSS version 23 (IBM Corporation, Armonk, NY, USA). An exploratory analysis was first conducted to verify the assumptions of normality and homogeneity. Data normality was assessed using the Shapiro–Wilk test (p>0.05), and homogeneity of variances was confirmed by Levene’s test (F=2.13, p=0.0976), indicating that the assumptions for parametric analysis were satisfied. After verification, the variables ΔE_00_, ΔWI_D_, cell viability, oxidative stress, and HP diffusion were analyzed by one-way ANOVA followed by Tukey’s post hoc test. A significance level of 5% was adopted for all analyses.

## Results

### Color analysis

#### ΔE00 (Figure 3A)

No significant differences were observed between 6%HP_MnO_BioS_LED (8.50±2.80) and 35%HP (6.48±1.81) groups (p=0.212). Both showed significantly higher ΔE_00_ values compared to 6%HP (3.24±1.19; p<0.001). Groups 6%HP_MnO_BioS (3.92±2.18) and 6%HP_LED (4.59±1.20) presented intermediate values, with no significant differences between them (p=0.674) or in comparison to the other experimental groups (p>0.05).

#### ΔWID (Figure 3B)

The 6%HP_MnO_BioS_LED group exhibited the highest ΔWI_D_ mean value (21.16±3.42), significantly higher than 6%HP (8.26±2.79; p<0.001) and 6%HP_MnO_BioS (9.45±3.15; p<0.001). The 35%HP group (16.33±4.04) showed intermediate results, not significantly different from 6%HP_LED (11.33±3.12; p=0.086) but higher than 6%HP (p=0.004) and 6%HP_MnO_BioS (p=0.009).

**Figure 3:**
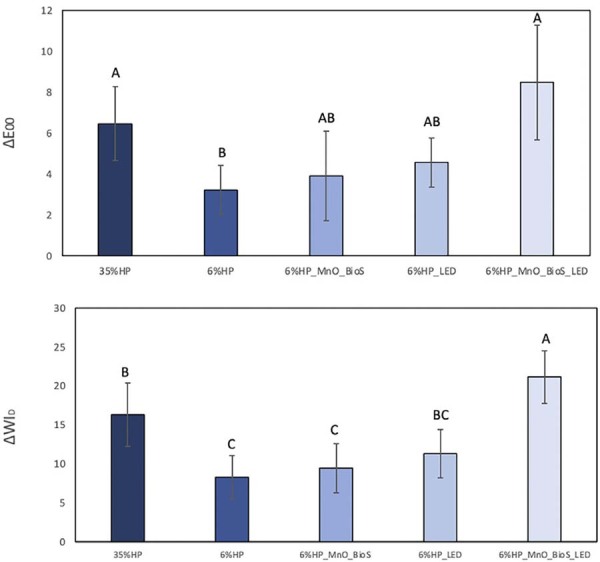
Mean and standard deviation of ΔWI_D_ (Figure 3A) and ΔE_00_ (Figure 3B) 24 hours after the last bleaching session (T_2_ – T_0_). Different letters indicate statistically significant differences according to one-way ANOVA and Tukey’s test.

## Cell viability

**Figure 4:**
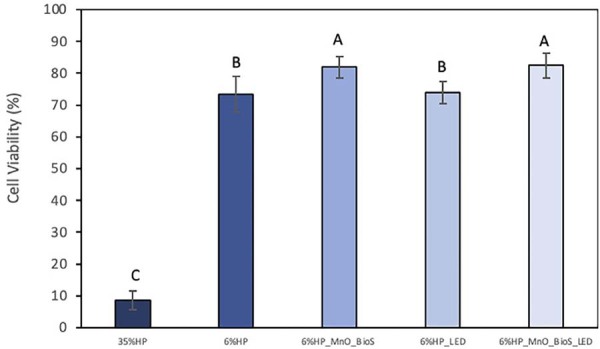
Mean values and standard deviation of cell viability (%)30 minutes after bleaching application. Different letters indicate statistical differences according to one-way ANOVA and Tukey’s test.

Figure 4 shows the mean values and standard deviation of cell viability (%) after 30 min of bleaching application. The experimental gels containing MnO_BioS, irradiated or not by LED, promoted the higher cell viability, while 35%HP caused lower cell viability among the groups (p=0.003). Groups 6%HP and 6%HP_LED presented intermediate values (p<0.0002).

## Oxidative stress

**Figure 5:**
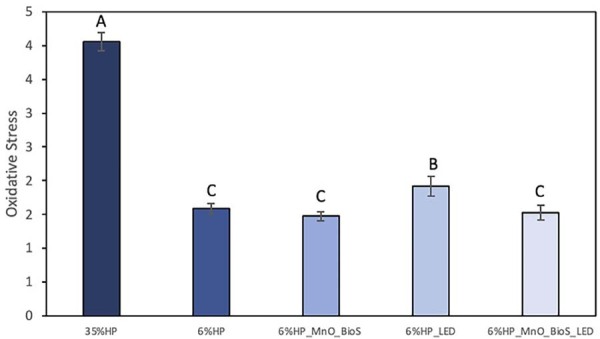
Mean values and standard deviation of oxidative stress (OxS) after a single treatment. Different letters indicate statistical differences according to one-way ANOVA and Tukey’s test.

Figure 5 shows the cells’ oxidative stress (OxS) determined by fluorescence values. 35%HP treatment induced the highest OxS, while 6%HP_LED had intermediate values (p=0.001***).*** Groups 6%HP, 6%HP_MnO_BioS, and 6%HP_MnO_BioS_LED exhibited the lowest OxS values among the groups (p=0.001), with no significant differences between them (p=0.943).

## Hydrogen peroxide quantification

**Figure 6:**
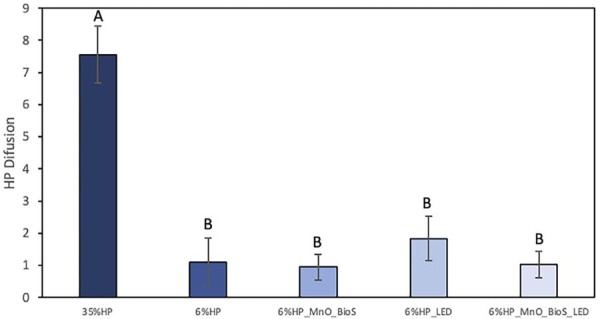
Mean values and standard deviation of HP diffusion (µg/mL). Different letters indicate statistical differences according to one-way ANOVA and Tukey’s test.

Figure 6 presents the results of HP diffusion in the extracts (DMEM and components of the bleaching gels that diffused through the disks). The 35%HP group showed the highest HP diffusion among the groups (p=0.0001), while the 6%HP groups exhibited lower diffusion values than the 35%HP group (p=0.0001), regardless of the presence of MnO_BioS or LED irradiation.

## Confocal fluorescence microscopy (live/dead assay)

**Figure 7:**
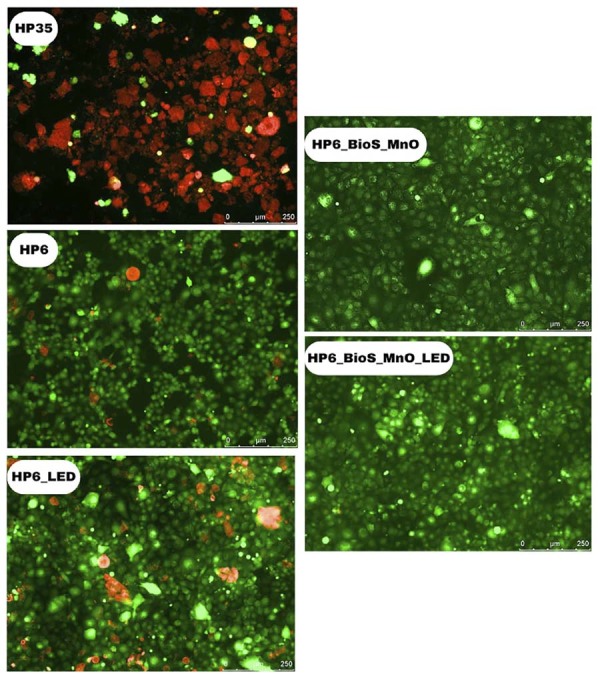
Confocal fluorescence microscopy with live/dead assay of MDPC-23 cells subjected to treatments with 35%HP, 6%HP, 6%HP_LED, 6%HP_MnO_BioS, and 6%HP_MnO_BioS_LED. Red fluorescence (Ethidium Homodimer-1, EthD-1) represents non-viable cells, and green fluorescence (Calcein AM - CA) represents viable cells.

Figure 7 shows representative confocal fluorescence microscopy images of pre-odontoblastic MDPC-23 cells subjected to the treatments. Cells treated with 35%HP demonstrated a decrease in viable cell clusters (green), an increase in necrotic cells (red), and a noticeable reduction in the total number of cells in culture compared to the other groups. When the HP concentration was reduced to 6%, an increase in the clusters of viable cells was noted, but with non-viable cells and an overall decrease in the total number of cells. Adding LED irradiation to the 6%HP treatment led to an increase in the concentration of non-viable cells and a reduction in viable cell clusters. The 6%HP_MnO_BioS combination, with or without LED, showed remarkable preservation of cell viability, with larger clusters of viable cells (green), a higher concentration of viable cells, and minimal or almost no necrosis compared to the other HP treatments.

## Discussion

The evaluation of color variation (ΔE_00_) revealed no significant differences between the 6%HP_MnO_BioS_LED and 35%HP groups. However, the 6%HP_MnO_BioS_LED group achieved a higher bleaching index (ΔWI_D_), surpassing the 35%HP group. These findings lead to the rejection of the first null hypothesis, as the addition of MnO_BioS enhanced the bleaching efficacy compared to the 6%HP gel, and only the 6%HP_MnO_BioS_LED group surpassed the color perception threshold of the 35%HP gel. This confirms that the combination of 6%HP with MnO_BioS, paired with violet LED irradiation, achieves a bleaching efficacy comparable to the commercial gel, highlighting the synergistic effect of MnO_BioS particles and violet LED light (405–410 nm). Furthermore, the gels used exceeded the perceptibility (PT) and acceptability (AT) thresholds (ΔE_00_ > 0.8 and 1.8, respectively).^18^ Clinically, this implies that the 6%HP_MnO_BioS_LED combination not only reached but exceeded the color perception and acceptability thresholds more than other groups, which highlights its potential for achieving effective and clinically relevant whitening results. This suggests that even with a lower concentration of hydrogen peroxide, the inclusion of MnO_BioS and violet LED light can significantly enhance the whitening outcome, providing a treatment option that is both effective and clinically perceptible. Consistent with these findings, a recent study^17^ demonstrated similar results, showing that the MnO-doped Biosilicate^®^ combined with violet LED promoted superior whitening efficacy and improved enamel safety compared to high-concentration hydrogen peroxide. The authors attributed this enhancement to the photocatalytic activity of MnO species, which accelerates the decomposition of hydrogen peroxide into reactive intermediates while maintaining pH stability and minimizing mineral loss.^17^

MnO_BioS acts as a redox catalyst in the decomposition of hydrogen peroxide (HP),^19,21^ and its activity is enhanced by violet LED irradiation (405–410 nm), accelerating the generation of highly reactive species such as OH and O₂-, which contribute to the bleaching process.^7,19^ Manganese oxides present semiconductor and photocatalytic behavior; excitation by near-violet visible light promotes charge separation (e-/h^+^) and facilitates the conversion of HP into reactive radicals.^15,20^ In clinical bleaching systems, light primarily serves as a coadjuvant that increases the rate of HP decomposition and chromophore oxidation, rather than directly generating radicals in isolation.^14,25^ The emission spectrum of violet LED light (≈405–410 nm) overlaps with the absorption bands of dental chromophores, promoting the photodegradation and fragmentation of pigmented organic molecules when combined with peroxide-based gels.^22-24^ Thus, the synergistic combination of MnO_BioS particles and violet LED irradiation enhances the bleaching efficacy of HP even at lower gel concentrations, with evidence showing improved performance and biocompatibility when MnO-enriched gels are photoactivated.^7^

This investigation revealed a direct relationship between cell viability and the presence of MnO_BioS in the bleaching hydrogel. The 35% HP gel showed the lowest cell viability, whereas reducing HP concentration to 6% significantly improved it. Hydrogels containing MnO_BioS further enhanced cell viability, regardless of LED irradiation. MnO_BioS, a silica-based bioactive glass-ceramic,^12^ interacts with HP to generate highly reactive hydroxyl (•OH) and superoxide radicals (O₂^•-^),^19,21^ accelerating HP decomposition into water (H₂O) and oxygen (O₂). The silica phase also acts as a nucleation center for hydroxyapatite formation^13^ and as an active surface capable of adsorbing and activating peroxide ions, facilitating radical generation. The MnO component, due to its redox activity, catalyzes HP decomposition through oxidation of Mn^2+^ to Mn^3+^, which serves as an additional oxidizing agent promoting radical formation.^9,19^ This catalytic process, analogous to Fenton-type reactions, contributes to a controlled reduction of reactive oxygen species (ROS) that mitigates cytotoxicity, thereby improving cell viability.^9^

The MnO_BioS groups and the 6% HP group reduced oxidative stress by nearly 40% compared to 35% HP. The HP6_LED group had intermediate results, suggesting that LED irradiation alone, in the absence of MnO_BioS, increased oxidative stress in the low-concentration gel. Nevertheless, all low-concentration bleaching gels presented lower oxidative stress than 35% HP. These findings indicate that the reduced HP content limits free H₂O₂ diffusion to the pulp. In addition, experimental groups containing MnO_BioS may display an extra ability to catalyze residual ROS and HP due to the redox cycling of Mn species.^9,19^ This antioxidant behavior decreases intracellular ROS, preventing cellular damage and enhancing cell viability, especially under bleaching conditions.^7,9^

The controlled release of mineral ions, such as calcium and phosphate, resulting from the interaction between the silica in MnO_BioS and the aqueous medium,^11,12^ may also contribute to the reduction of oxidative stress. These ions can promote remineralization of dentin and strengthen the dental structure, creating a more stable and favorable environment for pulp cells.^12,13^ The presence of these ions helps to maintain the pH^17^ and modulate the cellular response, which can directly affect the preservation of cell viability in contact with the bleaching gel. Additionally, the surface reactivity of MnO_BioS should be considered, as both the outer surface and the internal walls of the nanoporosity of these vitreous ceramics^11,12^increase the contact area with the environment, presenting antioxidant potential in cells and tissues. This means that MnO_BioS could “capture” and neutralize the excess HP generated by the decomposition of peroxide and also by the photocatalysis stimulated by violet LED, reducing the ROS that could come into contact with the pulp tissue.^7,17,19^ Furthermore, MnO_BioS can act as a buffering agent, supersaturating the medium with carbonated hydroxyapatite, contributing to the maintenance of an alkaline pH during bleaching.^12,17^ This is crucial since an acidic pH can promote the generation of ROS, increasing oxidative stress.^14^

The results also showed that the trans-enamel-dentin diffusion of HP is proportional to the concentration of the bleaching gel.^2,3^ Thus, the lowest HP diffusions were observed in the groups with 6% HP in their composition. Although not statistically significant, there was a tendency for a higher diffusion of HP to the pulp substrate when the gel was irradiated with violet LED in the absence of the bioactive compound.^7^

When MnO_BioS was added, the diffusion decreased [17]. When MnO_BioS interacts with the aqueous medium, a sequence of surface–solution interactions occurs.^11,12^ Initially, the surface of the Biosilicate^®^ particles undergoes repolymerization and reorganization of Si–O bonds, accompanied by the partial dissolution of surface components. This process forms a modified, denser surface layer with altered physicochemical properties, which acts as a barrier to hydrogen peroxide (HP) diffusion and reactive oxygen species (ROS) such as hydroxyl and superoxide radicals.^11,12^ This barrier reduces HP penetration even at early stages of interaction. Subsequently, a silica-rich (SiO₂) layer forms, further impeding HP diffusion by interacting with calcium and phosphate ions to create complexes and silicon networks.^11,12^ Finally, an amorphous calcium phosphate (CaO–P₂O₅) layer precipitates, a precursor to hydroxyapatite (HCA), confirming the bioactivity of MnO_BioS.^12^

This study employed standardized enamel/dentin discs to simulate the characteristic thickness of human lower incisors, teeth particularly susceptible to the adverse effects of bleaching. Cellular damage to the pulp correlates with HP concentration, enamel/dentin thickness, and gel application time.^2,3^

The Live/Dead assay images confirm that 35% HP may increase cell death. In contact with pulp cells, free HP triggers oxidative stress associated with lipid peroxidation, which inevitably leads to irreversible cellular damage.^2,3^ In this study, immortalized MDPC-23 cells, with an odontoblastic phenotype, were used to evaluate the potential trans-enamel-dentin cytotoxic effect of bleaching gels containing MnO_BioS. Odontoblasts are the first pulp cells to come into contact with components of dental materials that may diffuse through hard tissues and reach the pulp.^3^ Overall, this investigation showed that a considerable number of MDPC-23 cells were irreversibly damaged when the bleaching gel with 35% HP was applied to enamel/dentin discs, while groups containing MnO_BioS and/or lower HP concentrations showed improved viability.^7,17^

Despite the promising results, certain limitations need to be considered. Firstly, the cytotoxicity evaluation was conducted in *in vitro* models, which do not fully reflect the complexity of biological interactions in a clinical environment, where factors such as tissue inflammatory response, temperature variation during irradiation, intrapulpal pressure, and saliva dynamics can influence the results.^3^ The potential long-term toxicity of MnO_BioS, especially regarding diffusion and accumulation of metallic ions in the dental pulp, should also be addressed. Although the results indicate a reduction in oxidative stress and an improvement in cell viability, it is essential to further explore the effects of continuous use of MnO_BioS in bleaching treatments. Finally, the combination of 6% HP with MnO_BioS proved to be as effective as the commercial 35% HP gel, with lower cytotoxicity and potential for reducing oxidative stress.^7,9,17^
